# An Integrated Review of Carpal Tunnel Syndrome: New Insights to an Old Problem

**DOI:** 10.7759/cureus.40145

**Published:** 2023-06-08

**Authors:** Adekunle E Omole, Ayoola Awosika, Anosh Khan, Uzochukwu Adabanya, Nikhilesh Anand, Tirath Patel, Carolyn K Edmondson, Adegbenro O Fakoya, Richard M Millis

**Affiliations:** 1 Anatomical Sciences, American University of Antigua, Saint John, ATG; 2 College of Medicine, University of Illinois, Chicago, USA; 3 Emergency Medicine, Spartan Health Sciences University, Vieux Fort, LCA; 4 Community Medicine, Mercer University School of Medicine, Columbus, USA; 5 Pharmacology, American University of Antigua, Saint John, ATG; 6 Surgery, American University of Antigua, Saint John, ATG; 7 Clinical Medicine, American University of Antigua, Saint John, ATG; 8 Cellular Biology and Anatomy, Louisiana State University Health Sciences Center, Shreveport, USA; 9 Pathophysiology, American University of Antigua, Saint John, ATG

**Keywords:** median nerve, nerve entrapment, neuropathy, carpal tunnel, carpal tunnel syndrome

## Abstract

Carpal tunnel syndrome (CTS) is a common entrapment neuropathy characterized by pain, numbness, and impaired function of the hand due to compression of the median nerve at the level of the wrist. Although CTS can develop from repetitive strain, injury, or medical conditions, there are also congenital and genetic risk factors that can predispose individuals to the condition. With respect to anatomical factors, some individuals are born with a smaller carpal tunnel, which increases their susceptibility to median nerve compression. Variations in specific genes, such as those encoding proteins involved in extracellular matrix remodeling, inflammation, and nerve function, have also been linked to an increased risk for CTS. CTS is associated with a high cost of health care maintenance and loss of work productivity. Therefore, it is vital that primary care physicians fully understand the anatomy, epidemiology, pathophysiology, etiology, and risk factors of CTS, so they can be proactive in prevention, diagnosing, and guiding proper treatment. This integrated review also provides insights into how biological, genetic, environmental, and occupational factors interact with structural elements to determine who is most likely to acquire and suffer from CTS. Keeping health practitioners abreast of all the factors that could impact CTS should go a long way in decreasing the health care and socioeconomic burden of CTS.

## Introduction and background

**Figure 1:**
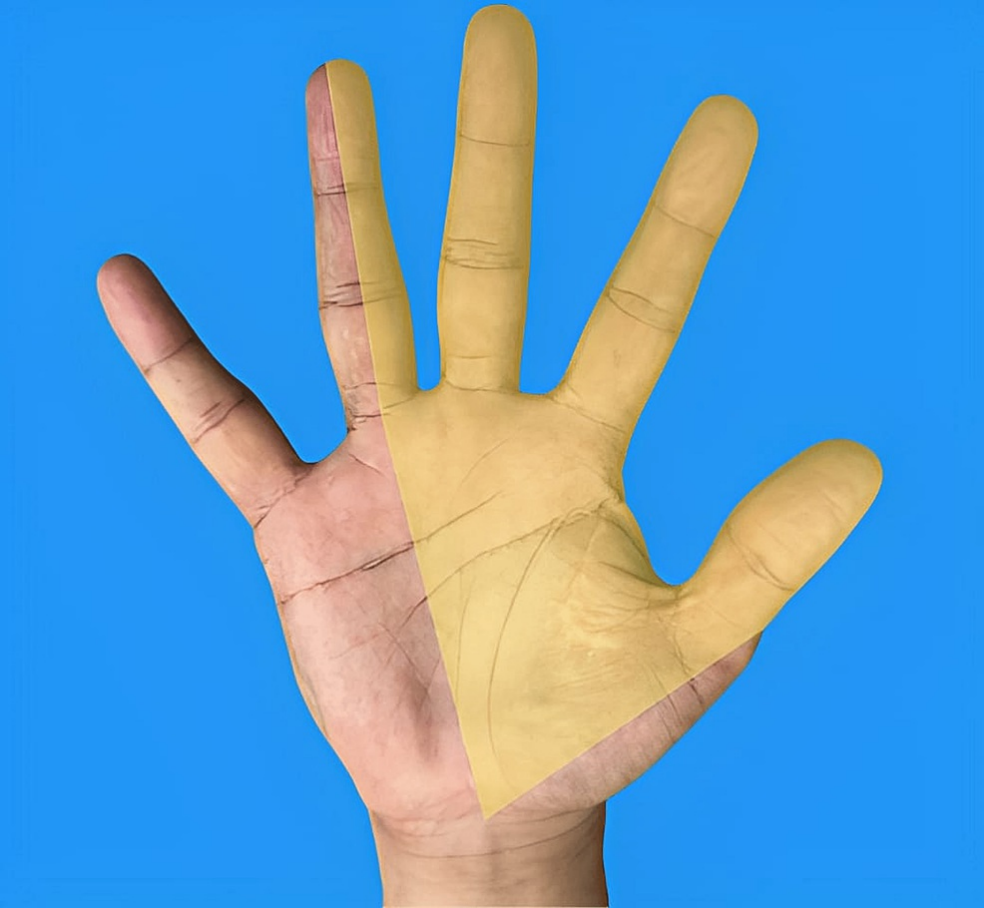
Median nerve distribution in the palmar aspect of the right hand This is an original image from the ultrasound lab department of the American University of Antigua

Carpal tunnel syndrome (CTS) is a common medical disorder affecting the general patient population. It is the most common peripheral nerve entrapment syndrome, accounting for approximately 90% of all focal entrapment neuropathies [1,2]. CTS occurs when the median nerve is compressed as it travels through the wrist, leading to symptoms like pain, numbness, and paresthesia in the palmar distribution of the median nerve in the hand, namely the thumb, index finger, middle finger, and the radial half of the ring finger (Figure 1). Similarly, pain, numbness, and paresthesia are experienced in the dorsal distribution of the median nerve in the hand, namely the dorsum of the distal halves of the same aforementioned lateral three-and-a-half fingers. With further progression of the disorder, weakness of the hand, poor grip strength, decreased fine motor coordination, and thenar muscle atrophy can occur. Initially, symptoms tend to be more common at night, but they become more constant with further compression.

In the United States, CTS is the most expensive upper extremity musculoskeletal disorder, with healthcare costs exceeding $2 billion yearly [3]. Most patients with CTS are industrial workers, females, and elderly persons who present to health practitioners for the first time. Consequently, it is important for physicians and allied health professionals to fully understand the syndrome so they can diagnose CTS and guide proper treatment. This review highlights the current state-of-the-art, as well as the research and knowledge gaps, with respect to CTS anatomy, epidemiology, pathophysiology, diagnosis, and management. Additionally, this study highlights the biological, genetic, environmental, and occupational factors interacting with the structural elements predisposing one to CTS.

## Review

Methodology

The literature search was done using web-based databases like Pubmed (the National Library of Medicine of the USA, which contains a database of medical and biomedical research literature), Google Scholar, EMBASE (the Excerpta Medica Database), Web of Science, and Scopus. MeSH terms used to guide the search strategy were formed using the patient/population, intervention, comparison, and outcome (PICO) criteria for evidence-based medicine investigations. The search terms adopted include 'carpal tunnel syndrome or median nerve compression or peripheral nerve entrapment syndrome', and 'environmental or genetic', and 'treatment modalities or rehabilitation', and 'quality of life'. Relevant articles were filtered based on set inclusion and exclusion criteria, followed by a manuscript review by two authors and a third party for any disagreements (Table 1).

**Table 1:** Summary of inclusion and exclusion criteria

Inclusion criteria	Exclusion criteria
Studies published in the last 15 years (2008-2023) in peer-reviewed journals	Exclusion of duplicates and editorials
Studies pertaining to median nerve compression or carpal tunnel syndrome	Article title and abstract that don't focus on genetic, occupational, and epigenetic of carpal tunnel syndrome
Studies that are written in English	Article title and abstract that don't focus on treatment modalities and rehabilitation in carpal tunnel syndrome

Anatomy of the carpal tunnel

**Figure 2:**
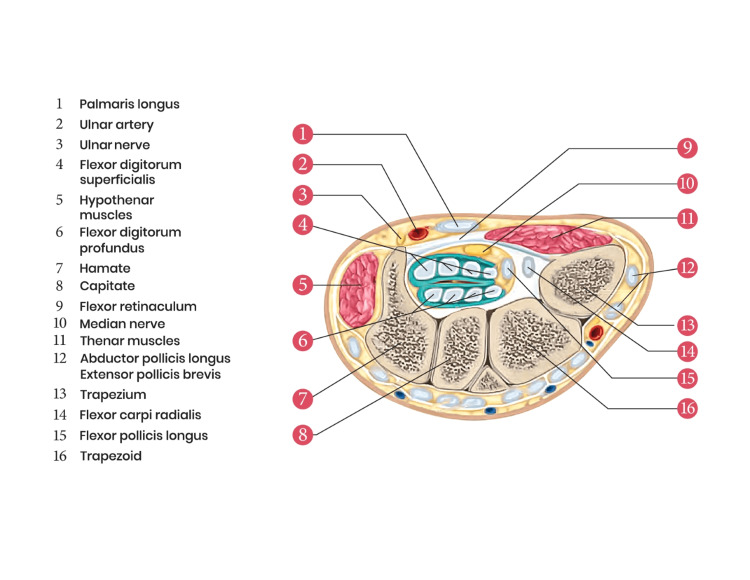
Anatomical diagram of the transverse section of a right wrist (at the distal row of the carpal bones) showing carpal tunnel and its contents This is an original image from the ultrasound lab department of the American University of Antigua

The carpal tunnel is formed at the proximal palmar area of the wrist at the junction of the forearm and the hand. This tunnel serves as a passageway for the extrinsic tendons and median nerve from the forearm to the hand. Superiorly, it is bounded by a sheath of tough connective tissue, the flexor retinaculum (transverse carpal ligament). The floor of this tunnel is formed by carpal bones. The eight carpal bones are arranged in two transverse rows (proximal and distal) and form an arch, which is convex on the dorsal side and concave on the palmar side. From the lateral to the medial side of the hand, the four carpal bones on the proximal row are scaphoid, lunate, triquetrum, and pisiform. In a similar order, the four carpal bones on the distal row are trapezium, trapezoid, capitate, and hamate. The flexor retinaculum is attached to the tubercle of the scaphoid bone and the ridge of the trapezium on the radial side of the hand. On the ulnar side of the hand, it is attached to the pisiform bone and the hook of the hamate. Running through this tunnel from the forearm to the hand are ten structures: nine long flexor tendons (flexor pollicis longus, four tendons of flexor digitorum superficialis, and four tendons of flexor digitorum profundus) and the median nerve. The tendon of the flexor carpi radialis (FCR) is located just outside the carpal tunnel. The median nerve is one of the major peripheral nerves of the upper limb [4]. It lies just below the flexor retinaculum as the most superficial structure of the carpal tunnel (Figure 2).

The median nerve passes from the anterior compartment of the forearm through the carpal tunnel into the wrist, where it forms branches to provide motor innervation to the thenar muscle group (abductor pollicis brevis, flexor pollicis brevis, and opponens pollicis), first and second lumbricals, and sensory innervation to the palmar surface of the thumb, index, middle and radial half of the ring finger and the dorsum of the distal halves of these fingers.

There are a few anatomical variations and anomalies of the median nerve in and around the carpal tunnel that is reported in the literature [5]. For instance, the high division of the median nerve proximal to the carpal tunnel, also known as the bifid median nerve, is a rare anatomic variation that may be associated with CTS [5,6]. It has an incidence of 0.8% to 2.3% in patients with CTS [6]. This variation may be associated with persistent median artery [7]. The bifid median nerve may facilitate compression of the median nerve in the carpal tunnel because of its increased cross-sectional area [6]. Another variation is noted in the recurrent (motor) branch of the median nerve, the branch that supplies the thenar muscles. This variation can be of several types [5]. We have the extra-ligamentous form (where the motor branch arises from the median nerve distal to the transverse carpal ligament on the radial side), sub-ligamentous form (where the motor branch arises within the carpal tunnel), and trans-ligamentous form (where the motor branch pierces the transverse carpal ligament on its course toward the thenar musculature) [5]. Additionally, the motor branch may take off from the ulnar, radial, anterior, or central part of the median nerve [5]. These differences illustrate the variable motor symptoms in cases of severe compression on the median nerve. Another variation, though rare, is the positioning of the ulnar nerve in the carpal tunnel [5]. In the event of its occurrence, patients present with CTS associated with symptoms of ulnar nerve compression, including hypothenar muscle wasting and paresthesia involving the little finger and the ulnar half of the ring finger.

In addition to anatomical variations related to the nerves in the carpal tunnel, vascular, tendinous, and muscular anatomical variations that possibly increase the risk of CTS have been reported in the literature. Examples are persistent median artery in the carpal tunnel (vascular anomaly), tendinous connection between the flexor digitorum profundus tendon of the index finger and the flexor pollicis longus tendon (tendinous anomaly), and presence of accessory palmaris longus muscle belly in the carpal tunnel [5]. By occupying the space in the carpal tunnel, these variations may increase the risk of CTS by causing significant constriction of the carpal tunnel and compression of the median nerve [5].

Awareness of these anatomical variations by physicians and surgeons is essential both during clinical examinations and during carpal tunnel release surgery. The symptoms for CTS may vary due to the variation in the anatomy. Thus, the lack of awareness of these variations may lead to misdiagnosis. If unrecognized, these variations can also increase the risk of iatrogenic injury to the involved structures leading to unfavorable surgical outcomes [5]. Generally, CTS may result from any lesion that significantly narrows the size of the carpal tunnel, which causes the swelling of the synovial sheaths of the long flexor tendons or increases the cross-sectional area of the median nerve [2].

**Figure 3:**
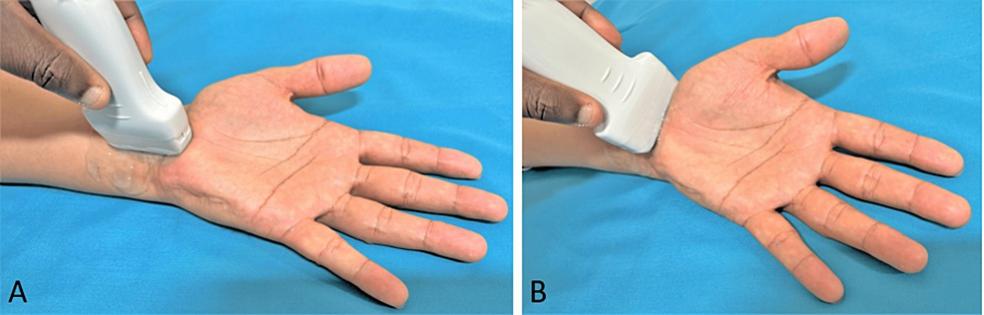
Ultrasound transducer placement technique; (A) longitudinal imaging, (B) transverse imaging This is an original image from the ultrasound lab department of the American University of Antigua

**Figure 4:**
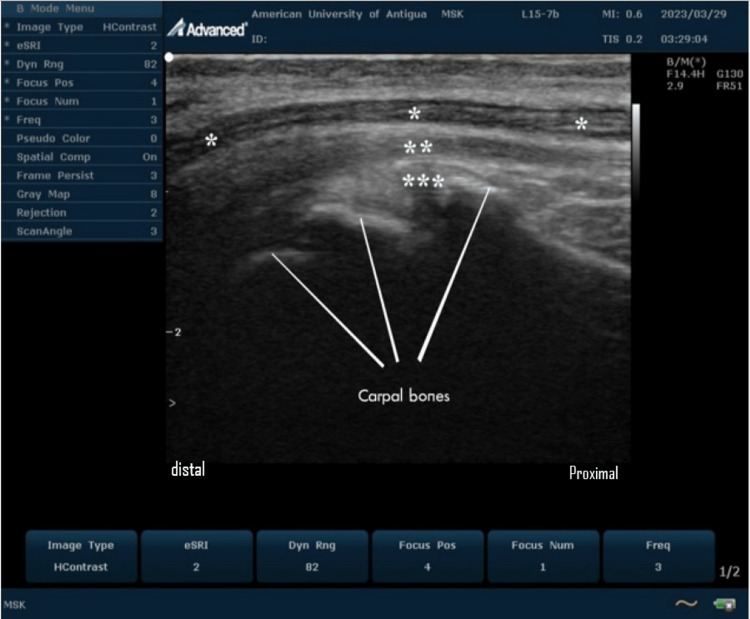
Ultrasound imaging of the normal carpal tunnel at the volar aspect of the wrist On this longitudinal scan, the median nerve (*) is seen overlying the relatively hyperechoic superficial (**) and deep (***) flexor tendons of the wrist. The median nerve appears as a hypoechoic honeycomb fibrillar structure. This is an original image from the ultrasound lab department of the American University of Antigua

**Figure 5:**
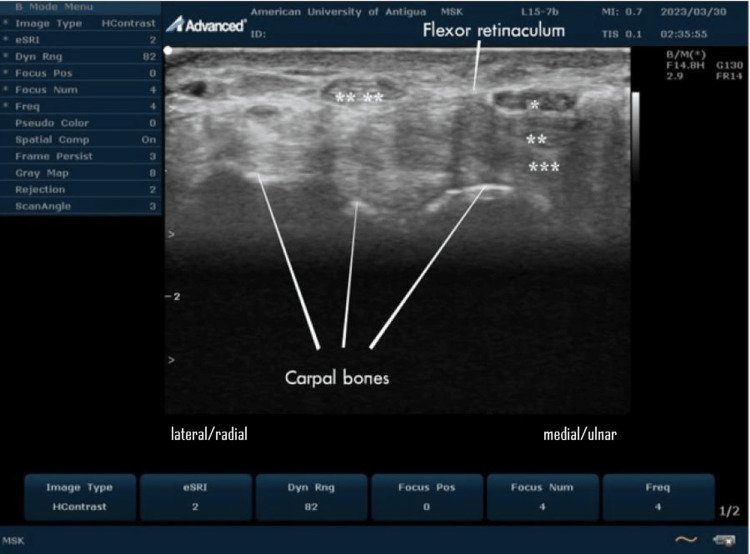
Ultrasound imaging of the normal carpal tunnel at the volar aspect of the wrist On this transverse scan, the median nerve (*) is seen overlying the relatively hyperechoic superficial (**) and deep (***) flexor tendons of the wrist. The hypoechoic oval median nerve is located deep in the hyperechoic flexor retinaculum and has a characteristic cyst-like appearance. The tendon of flexor carpi radialis (****) is just outside the carpal tunnel. This is an original image from the ultrasound lab department of the American University of Antigua

In real-time ultrasound of the carpal tunnel at the wrist, the median nerve usually appears as a hypoechoic oval structure with a honeycomb appearance just deep to the flexor retinaculum (Figures 3, 4, 5).

Epidemiology

The incidence and prevalence of CTS vary widely in the literature. Worldwide, an estimated 4-5% suffer from CTS, with the most liable population being elderly individuals aged between 40 and 60 years [8,9]. The UK General Practice Research Database in 2000 revealed that the prevalence of CTS was 88 per 100,000 in males and 193 per 100,000 in females showing that CTS is more common amongst women as compared to men [10,11]. CTS is a work-related musculoskeletal disorder caused by strain and repeated movement; thus, it is quite common among manual workers. The associated work absence and healthcare cost is a significant socioeconomic burden to the UK economy [10]. In the United States, the incidence of CTS is one to three persons per 1000 subjects per year, with a prevalence of 50 cases per 1000 subjects in the general population [2]. In South Africa, CTS most commonly affects White people and appears to be rare in Black South Africans [12]. The rate of CTS is two to three times more common among the White US Navy personnel compared to their black counterparts [13]. The median work time lost following CTS is 25 days, and healthcare costs in the United States exceed $2 billion per year [3,14]. This makes CTS a significant driver of overall workers' compensation costs, and understanding of CTS-associated disability is crucial for minimizing the undesirable effect of the ailment on workers and employers [14].

Etiology and risk factors

Most cases of CTS are idiopathic [15]. Several risk factors and diseases associated with this medical condition have been identified. These include female gender [4], genetic predisposition [2], aging [11], race (White people are two to three times more prone to get affected than Black people) [2], obesity [4,16], alcoholism [16], drug toxicities and exposure to toxins [16]. The peak age incidence in women is 45-54 years [15,16]. If a woman has not experienced the symptoms of CTS in her middle-aged years, she is less likely to experience them for the first time in old age [4]. Contrastingly, the incidence in men seems to increase with age [4]. Obese individuals are 2.5 times more likely to be diagnosed with CTS compared to non-obese ones [17]. Trauma (like fracture of the wrist) and diseases that cause inflammation may increase the volume within the carpal tunnel and thus lead to CTS [4]. A good example is rheumatoid arthritis, where the resulting pannus formation or synovitis can lead to an increase in carpal tunnel pressure. Additionally, space-occupying lesions in the carpal tunnel like tumors, lipoma, ganglion cysts, infection, and scar tissue, can lead to compression of the median nerve [16].

Occupational interactions with carpal tunnel syndrome

Numerous studies have shown an association between CTS and occupational activities. A review of these studies reveals a positive association between CTS and occupations involving highly repetitive wrist motion, use of vibratory tools, increased hand force, and prolonged or repetitive flexion/extension of the wrist [4,18-21]. For example, jobs involving food processing and packaging, forestry, stone carving, slaughterhouse, and textile work [4]. Most patients have often associated the onset of CTS with excessive computer keyboarding and mouse use. Surprisingly, up to date, the literature shows no clear evidence to support this association [18,22-23]. In the systematic review published by Thomsen et al. in 2008 and Mediouni et al. in 2014, it was concluded that some certain work circumstances involving computer mouse use may increase the incidence of CTS [22,23]. Although carpal tunnel pressure increases with computer keyboarding and mouse use, the pressure is still below harmful levels [22,23]. Thus, keyboarding and mouse use should be viewed as aggravating factors for CTS rather than as risk factors.

Risk factors at the workplace that showed an increased risk for CTS were high job strain, while social support appeared to be protective. The inverse relationship between CTS incidence and years worked among recent hires may indicate that there is an existence of a healthy worker survivor effect from the study population [24]. In this study, women had an increased hazard ratio, but there was no statistically significant increased risk for CTS in women (hazard ratio (HR)=1.30; 95% CI 0.98 to 1.72). Furthermore, the incidence of CTS correlated with linear increments in age and body mass index (BMI). High levels of strenuous work-related activity increased the risk of CTS (HR=1.86; 95% CI 1.11 to 3.14), social support was a protective variable (HR=0.54; 95% CI 0.31 to 0.95), and there was an inverse relationship between years worked, with the greatest incidence in the first 3.5 years of work (HR=3.08; 95% CI 1.55 to 6.12) [24].

In summary, CTS is a particularly common disease in the general population, and the relationship of CTS to occupational activities, either independently or in combination with other factors, has been well documented by epidemiological data. Work disability among people with CTS is common, and for those with CTS, working environments characterized by repetitive bending of the hand or wrist may increase the risk of work disability associated with CTS [25,26]. Prevention of severe disability in CTS patients should target forceful exertions and involves a decrease of both biomechanical and logistic work stressors and the development of comprehensive intervention strategies for those prone to such work disability. There is a clear need for further research to understand the risk factors associated with work disability among those with CTS.

Hormonal interplay with carpal tunnel syndrome

Hormonal factors play a role in the gender differences observed in CTS. Accordingly, the incidence of CTS is higher in pregnancy, breastfeeding women, women in their first menopausal year, and women on oral contraceptive pills or hormone replacement therapy [4,27-28]. However, oophorectomy reduces the incidence of CTS [4]. Some CTS cases are linked with endocrine disorders such as hypothyroidism, acromegaly, and diabetes mellitus [4]. Theoretically, CTS in hypothyroidism can be attributed to the resulting myxedema, which may cause the deposition of mucopolysaccharides and water within both the perineurium of the median nerve and the tendons of the carpal tunnel [28]. With acromegaly, the hypersecretion of growth hormones causes excessive growth of the soft tissues and bones around the carpal tunnel, which may lead to compression of the median nerve [28].

Genetic factors, genetic polymorphisms, epigenetics, and amyloidosis in carpal tunnel syndrome

The genesis of CTS may also be influenced by genetic factors. It is believed that there are three distinct processes - the production of collagen, the breaking down of collagen, and the defense against oxidative stress in connective tissue - that may contribute to a person's inherited likelihood of developing CTS [29]. These pathways are regulated and controlled by several gene groups that may be involved in the development of CTS. CTS is attributed to variations in collagen gene variants like - COL1A1, COL5A1, and COL11A1 - which result in the production of various subtypes of collagen and cause changes in the tendons and other connective tissue components' (CTC) mechanical properties [29]. Matrix metalloproteinases (MMPs), which also modify the collagen inside CTC, have been studied for their potential significance in the risk of CTS development [29]. Next, it was discovered that the variations of the genes that code for glutathione S-transferase (GST) production are related to the etiology of CTS [29].

Amyloidosis is another factor that contributes to the etiology of CTS [30]. Amyloidosis is a localized or systemic disease characterized by extracellular deposition and accumulation of aberrant fibrillar insoluble proteins known as amyloid in organs and tissues of the body. Once considered rare and incurable, amyloidosis is now increasingly recognized by clinicians as an important cause of heart failure [30]. Although bilateral CTS has long been recognized to be a manifestation of light chain amyloidosis (AL), recent studies have found that this condition is even more common in transthyretin amyloidosis (ATTR), especially wild-type transthyretin-related amyloidosis (ATTRwt), occurring in 20%-60% of patients, usually 5-10 years before the onset of cardiac manifestations [30].

Collagen gene variants

It has been suggested that changes in the basic properties of collagen, such as its elasticity and durability, could affect the pressure in the carpal tunnel (CT) and lead to median nerve compression. Table 2 summarizes numerous research studies that have revealed a connection between variations in genes responsible for collagen production and degradation, and their potential role in causing CTS. As a result, genetic factors should be considered in individuals who may be at risk of developing CTS.

**Table 2:** Collagen gene variants, their mechanism, and how they relate to CTS CTS - carpal tunnel syndrome, CTC - connective tissue components

Gene Variant	Mechanism	Relation with CTS
Presence of the COL11A1 gene's T allele (rs1676486) [31,32].	Decreased formation of the α1(XI) collagen chain.	The etiology of CTS may be related to type XI collagen production.
The existence of variations (rs3753841, rs1676486 and rs1746744) in the T-C (AGGG) section of the COL5A1 and COL11A1 genes [31,33].	Altered type V and XI collagen formation as a result of abnormal mRNA stability	The cause of CTS is because of the mechanical changes that alter the properties of tendons and other CTC inside the CT
A variation in the COL11A1 gene at position rs3753841 that involves a change from T to C at a single nucleotide site [32].	The change in exon 52 results in a switch from leucine to proline in the α1(XI) chain at position 1323, causing a non-synonymous substitution.	This can impact the formation of type XI collagen, causing disruptions to the structure and performance of newly formed collagen fibers. These disruptions within the CT, could potentially contribute to the development of CTS.
A variation in the COL11A1 gene at position rs1676486 that involves a change from T to C at a single nucleotide site [32].	This variation in exon 62 causes a change in the amino acid from proline to serine in the α1(XI) chain at position 1535, leading to a non-synonymous substitution.	This can impact the formation of type XI collagen, causing disruptions to the structure and performance of newly formed collagen fibers. These disruptions within the CT, could potentially contribute to the development of CTS.
The COL1A1 gene has a Minor variant of the T allele (designated as rs1800012, with the notation G/T), which codes for type I collagen's α1 chain [33,34].	In intron 1 of COL1A1's Sp1 binding site, replacing tyrosine with a guanine nucleotide increases the transcription factor Sp1's binding affinity, leading to heightened COL1A1 gene expression and overproduction of type I collagen homotrimers comprised of three α1 chains.	It is believed that a rise in the levels of type I collagen homotrimers in tendons and other CTC may alter their mechanical properties and make them more susceptible to injury. This alteration could also play a role in the generation of higher pressure in the CT.

Matrix metalloproteinase (MMP) genes

Studies have demonstrated that the variants of MMP genes hold significance in the remodeling of connective tissue through the degradation of collagen fibrils [29]. To examine the possibility of a connection between different variations of MMPs and the likelihood of developing CTS, research was initiated due to MMPs' potential impact on the stability of soft tissue and its components (such as tendons and ligaments). Four MMP variations were especially suspected because of their potential activities, including the capacity to break down different kinds of collagen and because of previously known connections with other musculoskeletal illnesses [29]. The additional guanine nucleotide at position 1607 bp can either be present (GG) or absent (G) in the MMP1 rs1799750 (G/GG) variation [29]. The GG variant results in elevated expression of the MMP1 gene through the establishment of an Ets binding site, whereas the allele lacking the extra G nucleotide does not [29]. The variant of MMP12 rs2276109 (A/G) affects the connection of the AP-1 protein, which acts as a transcription factor and manages the manifestation of MMP12 [29]. The variations in the MMP3 gene (rs679620) and MMP10 gene (rs486055) result in a single-nucleotide alteration which leads to a modification in the amino acids that are formed by these genes [29,35-36]. The precise effects of these alterations in amino acids are still undetermined, but prior studies have hinted at a possible link to diseases affecting tendons and ligaments, a heightened likelihood of sustaining injuries in these areas, and the emergence of CTS.

Glutathione S-transferase (GST) gene variants

Idiopathic CTS is believed to be caused by oxidative stress caused by an excessive amount of free oxygen and hydroxyl radicals in the tendons and ligaments' sub-synovial soft tissue [37,38]. GST isoenzymes are a large group of related proteins that aid in protecting cells from the harmful effects of reactive oxygen species by neutralizing their byproducts. It is formulated that GST genes may serve a defensive role against the development of systemic inflammatory diseases and cancers due to oxidative stress [39]. Deletions are the most widespread changes in the GSTM1 and GSTT1 genes, resulting in reduced enzyme function. Another common alteration is the GSTP1 Ile105Val, where the Ile105Val/Val (AG) and Ile105Val/Val (GG) versions have a decreased level of enzyme activity compared to the Ile105Val/Val (AA) version [40]. Correlations between specific GST enzyme variations and illnesses like rheumatoid arthritis, diabetes, and cancers that result from reactive oxygen species have been reported [39]. These findings have prompted researchers to explore potential connections between GST gene variants and the risk of developing CTS. Eroğlu et al. reported such connections in their study of the incidence of three GST variants - GSTM1, GSTT1, and GSTP1 Ile105Val - in Turkish populations of CTS patients and controls (n=140 and n=97, respectively). They found a statistically significant (p = 0.01) higher incidence of GSTM1-null variant in CTS patients compared to the healthy controls. Additionally, the GSTM1-null variant was associated with a roughly twofold increase in the risk of CTS. The combination of GSTM1-null and GSTT1 variants was also more prevalent in CTS patients, although it did not increase the risk of the development of CTS. Finally, they reported a statistically significant association between higher clinical severity of CTS in patients with the GSTP1 Ile/Val and Val/Val variants compared to GSTP1 Ile/Ile, which was more frequent in patients with a clinically milder disease [38]. They concluded in their report that further research investigating the potential role of GST variants in the development of CTS is justified.

Amyloidosis

The carpal tunnel with a constant volume of 5 ml, is encased on three sides by the carpal bones and on the front side by the flexor retinaculum. In amyloidosis, the buildup of amyloid in the tendons and nearby tissues decreases the space within the tunnel, causing compression of the median nerve, leading to long-term CTS [41]. Previous studies have demonstrated amyloid deposition in carpal ligaments ranging from 10%-35%, with increased rates in men and advancing age [42]. Although AL and other amyloid types were occasionally detected by pathology, most recent studies of CTS have found ATTRwt deposition [43].

Milandri A et al. reported a higher prevalence of cardiac involvement in hereditary transthyretin-related amyloidosis (ATTRm) and ATTR wild-type (ATTRwt) than anticipated [30]. In ATTRm and ATTRwt, the prevalence was 14% and 25%, respectively, similar to the general population with AL. The study discovered that CTS increases the likelihood of cardiac amyloidosis occurring in individuals who have either ATTRwt or ATTR with specific genetic mutations. Furthermore, the occurrence of CTS has been correlated with a more developed form of heart disease and occurs prior to the identification of hereditary transthyretin amyloidosis with cardiomyopathy (ATTR-CA) by a period of five to nine years, especially in males [30].

Although it is widely recognized that there is an epidemiological connection between CTS and coronary artery disease, the exact cause is not yet understood. The reason for the specific involvement of these two tissues and the time lag between them is still a mystery; however, it is speculated that repetitive physical strain on the tendons and heart muscle may contribute to the formation of fibrils and/or tissue invasion [44].

Charcot-Marie-Tooth (CMT) disease and CTS

CMT disease is one of the most common inherited neurological disorders, affecting approximately one in 2,500 individuals [45]. CMT is characterized by progressive muscle weakness, sensory loss, and foot deformities. Berciano et al. conducted a retrospective study on 172 CMT patients and found that 25% of them had clinical and electrophysiological evidence of CTS [46].

CMT affects the structure and function of peripheral nerves, making them more vulnerable to compression at anatomical sites such as the carpal tunnel [46]. Muscle atrophy and weakness in CMT patients may lead to changes in hand use patterns, increasing the risk of median nerve compression. CMT patients are known to experience multiple sites of nerve compression, a phenomenon known as "double crush syndrome". This could potentially increase the risk of CTS in CMT patients.

Ehlers-Danlos syndrome (EDS) and CTS

EDS is a group of heritable connective tissue disorders characterized by hypermobility, skin elasticity, and tissue fragility. Gharbiya et al. conducted a study on 46 patients with EDS and found that 15% of them reported symptoms suggestive of CTS, though electrophysiological confirmation was not performed [47]. The association between EDS and CTS can be explained by several mechanisms. EDS patients often exhibit joint hypermobility, which can lead to joint instability and increased pressure on the median nerve within the carpal tunnel [48]. Connective tissue fragility in EDS patients can result in laxity of the soft tissues surrounding the carpal tunnel, potentially causing nerve compression [49]. EDS patients may be more susceptible to repetitive strain injuries due to joint instability and muscle weakness, increasing the risk of developing CTS [50].

Pathophysiology

As discussed in the section above, CTS is associated with many epidemiological risk factors, including genetic, social, demographic, occupational, and medical. An interplay between these factors eventually leads to the development of CTS. The pathophysiology of CTS is related to a combination of two processes: compression and traction mechanisms [2].

The compression mechanism results from an increase in carpal tunnel pressure [2]. Normally, in a healthy subject, the pressure in the carpal tunnel ranges between 2.5 to 13mmHg [18,51]. However, in CTS patients, the pressure may rise to critical levels above 20-30mmHg [18]. The abnormally high carpal tunnel pressure leads to obstruction to venous outflow, back pressure, increasing local edema, and eventually, ischemia of the median nerve [2]. The latter results due to the compromise of the blood flow to the endoneurial capillary system. The resulting alteration in the blood-nerve barrier interferes with the structural integrity of the median nerve, leading to demyelination and axonal degeneration [2]. The traction mechanism is due to repetitive traction and wrist movements. Flexion and extension of the wrist can cause eight- and ten-time increase in the carpal tunnel's fluid pressure, respectively [52]. Thus, repetitive wrist movements will further worsen nerve damage in CTS patients. Accordingly, splinting of the wrist to hold it in a neutral position is widely considered the first line of treatment for mild to moderate CTS cases.

Diagnosis

The diagnosis of CTS involves a combination of a thorough history and physical examination in conjunction with adjunctive tests like electrodiagnostic studies and ultrasonography [53].

History

A detailed history is a very vital component of the diagnostic workup toward the identification of CTS in a patient. With regards to the symptoms, the clinician should ask the patient about their duration, frequency, location, character, radiation (e.g., "Do symptoms radiate from the shoulder?"), progression (better, worse, or stable), timing (night, day, or both), frequency, severity, aggravating factors (certain positions or movements), relieving factors (ice, rest, shaking/flicking the hand and wrist, or use of splints). The shaking or flicking of the hands to provide relief from CTS symptoms is known as the flick sign. The flick sign is 93% sensitive and 96% specific [53]. Additionally, the clinicians should ask about the patients' lifestyle/activities, whether they use vibratory tools for their tasks, predisposing factors, and any comorbidities such as diabetes, obesity, pregnancy, or inflammatory arthritis.

History may also reveal the classic features of CTS, that is, pain, numbness, and paresthesia in the distribution of the median nerve, namely the thumb, index finger, middle finger, and the radial half of the ring finger. Numbness existing in the fifth finger or extending largely to the dorsum of the hand may suggest other diagnoses (Table 3). Additionally, there can be associated history of decreased strength with pinching or grip, with patients reporting that their hands fall asleep or things slip through their fingers unintentionally. Patients may describe difficulty in holding objects, buttoning a shirt, or opening jars. They may also report that the symptoms are worse at night and are associated with certain activities that fully flex/extend the wrist, such as driving, holding a telephone, reading newspapers, or painting.

**Table 3:** Differential diagnosis of carpal tunnel syndrome

Condition	Characteristics	Reference
Arthritis of the wrist	Pain, swelling, stiffness, and limited motion at the wrist. Radiographic findings (i.e., joint space narrowing and osteophytes in osteoarthritis; erosions and deformity in rheumatoid arthritis)	[54]
Carpometacarpal osteoarthritis of the thumb (thumb arthritis)	Pain, swelling, stiffness, and limited motion at the base of the thumb. Radiographic findings (i.e., joint space narrowing & osteophytes). Positive grind test.	[55,56]
Cervical radiculopathy (C6)	Neck pain, numbness in thumb and index finger, weakness of biceps and wrist extensors. Positive Spurling test or shoulder abduction test.	[57]
De Quervain tendinopathy	Pain and tenderness at the base of the radial side of the thumb. Positive Finkelstein test, Eichhoff test, or wrist hyperflexion and abduction of the thumb (WHAT) test.	[58,59,60]
Diabetic peripheral neuropathy	Numbness, tingling, or loss of sensations in the feet or hands, burning or shooting pain in the feet or hands ('stocking and glove' distribution). History of diabetes mellitus.	[61,62]
Pronator syndrome (Median nerve compression at elbow)	Pain and tenderness at the proximal forearm, sensory loss on palmar aspects of thumb, index, middle, and half of ring fingers, weakened wrist flexion, ulnar deviation of the wrist, loss of forearm pronation, weakened movement of the thumb, thenar muscles atrophy, loss of flexion at the joints of the index and middle fingers (Benediction hand). Positive Pronator compression test.	[63]
Raynaud syndrome	Discoloration, pain, numbness, and sensation of cold in the hands. Symptoms related to exposure to cold temperatures.	[64]
Ulnar compressive neuropathy	Paresthesia and/or numbness in the palmar and dorsal part of the ring and little fingers (claw hand at rest), loss of finger adduction/abduction, loss of thumb adduction. Positive Tinel sign at the elbow or wrist (Guyon canal). Positive Froment’s sign test.	[65]

Physical examination

This involves a complete examination of the upper limb, including the neck, shoulder, elbow, and wrist, to exclude other neurologic or musculoskeletal diagnoses. Initial inspection of the hand and wrist may reveal evidence of precipitating factors such as arthritic changes and other signs of prior injuries like abrasions, ecchymosis, deformities, swelling, and other skin changes. Inspection can also reveal wasting and atrophy of the muscles of the thenar eminence. A sensory examination may show a lack of two-point discrimination and abnormalities in sensation on the palmar aspect of the first three digits and radial one-half of the fourth digit of the affected hand. This will help to localize the symptoms to the median nerve distribution. A motor examination may reveal wasting of the thenar eminence muscle groups and weakness of thumb abduction and opposition. In clinical practice, the physical examination includes some special provocative tests with varying degrees of sensitivities and specificities [53]. Nevertheless, these tests are easy to perform, and a combination of positive findings from them can increase the possibility of CTS [53,66].

Median Nerve/Carpal Tunnel Compression Test (Durkan's Test)

**Figure 6:**
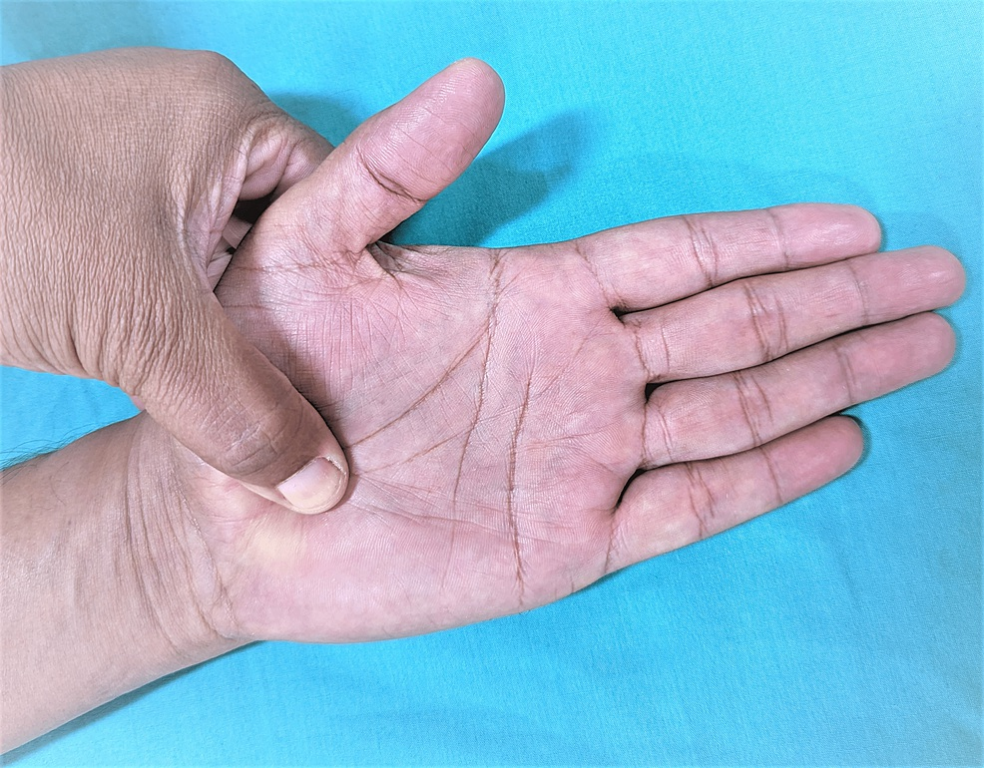
Median nerve compression test This is an original image from the ultrasound lab department of the American University of Antigua

It is considered the best of all the provocation tests for CTS [2,16]. This test has a sensitivity and specificity of 64% and 83%, respectively [53]. The examiner applies firm pressure directly over the carpal tunnel for 30 seconds. A positive test is demonstrated when the symptoms (pain, numbness, and paresthesia) are reproduced in the distribution of the median nerve (Figure 6).

Phalen's Maneuver

**Figure 7:**
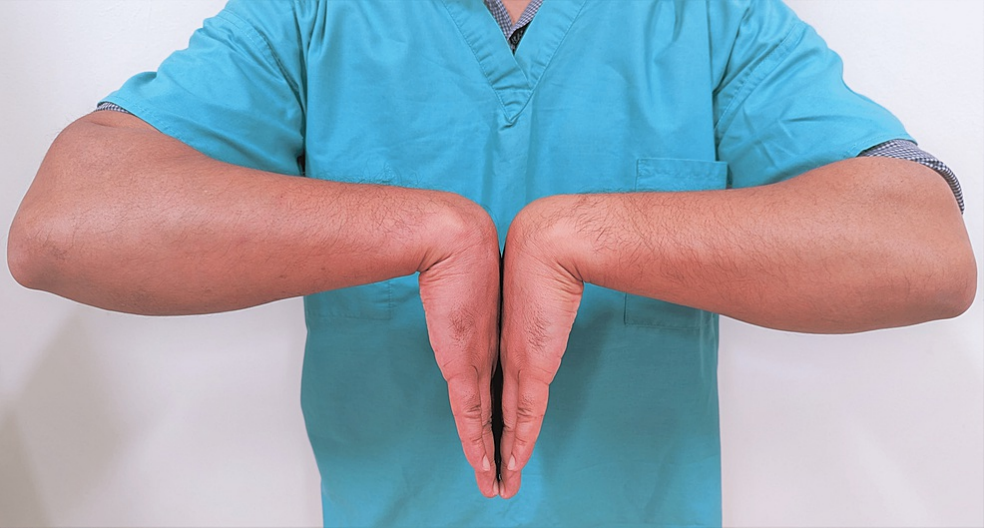
Phalen's test This is an original image from the ultrasound lab department of the American University of Antigua

This maneuver has a sensitivity and specificity of 57-68% and 58-73%, respectively [53]. The patient fully flexes both wrists to 90 degrees by placing the dorsal surfaces of both hands together for 60 seconds. A positive test is demonstrated when the symptoms are reproduced in the distribution of the median nerve (Figure 7).

Reverse Phalen's Maneuver (Prayer Test)

**Figure 8:**
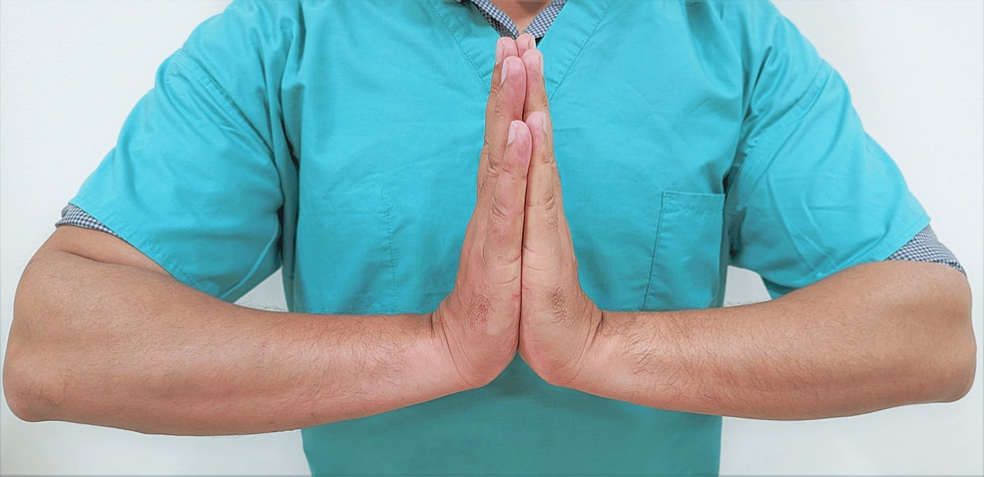
Reverse Phalen's test This is an original image from the ultrasound lab department of the American University of Antigua

The patient extends both wrists by placing the palmar surfaces of both hands together for 60 seconds, as if praying. Again, a positive test is demonstrated when the symptoms are reproduced in the distribution of the median nerve (Figure 8).

Tinel Sign

**Figure 9:**
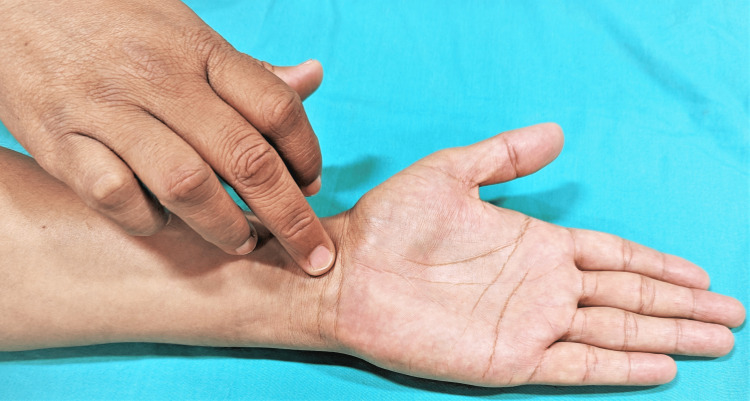
Tinel sign This is an original image from the ultrasound lab department of the American University of Antigua

It is 36-50% sensitive and 77% specific [53]. The examiner percusses/taps the volar surface of the patient's wrist over the carpal tunnel. A positive test is demonstrated when the symptoms are reproduced in the distribution of the median nerve (Figure 9).

Adjunctive tests

In a classic case of CTS, diagnosis is clinical (history and physical examination). Nonetheless, electrodiagnostic studies can assist in confirming the diagnosis, especially in atypical cases. Ultrasonography has been shown to be useful in diagnosing CTS as well.

Electrodiagnostic studies

Electrodiagnostic or electrophysiological studies involve nerve conduction studies (NCS) and electromyography (EMG). With NCS, you can measure the speed and strength of impulses propagated through the length of a peripheral nerve. Thus, it can confirm CTS by revealing impaired conduction through the median nerve. EMG records and analyzes electrical activity in the muscles. It can reveal pathological changes in the muscles innervated by the median nerve. However, electrodiagnostic studies have their limitations. For CTS, electrodiagnostic studies have a sensitivity and specificity of 56% to 85% and 94% to 99%, respectively [67]. False positives and negatives have been reported in electrodiagnostic studies. Witt et al., in 2004, revealed that NCS results were normal in up to one-third of patients with mild CTS [68]. Thus, electrodiagnostic studies are not a perfect gold standard for the diagnosis of CTS and cannot replace a clinical examination. However, in combination with clinical examination, they can serve as a powerful confirmatory tool for the diagnosis of CTS. Most importantly, electrodiagnostic studies can help in ruling out other neurologic diagnoses and determining the severity of CTS. Limited evidence exists in support of a correlation between findings from electrodiagnostic studies alone and functional recovery from CTS following carpal tunnel release surgery [69]. This limits the prognostic use of electrodiagnostic studies. However, if a repeat NSC test after the carpal tunnel release surgery shows improvement in median nerve function, this can help to reassure the patient. The American Academy of Orthopedic Surgery (AAOS) guidelines for diagnosing CTS recommend that the physician obtain electrodiagnostic studies if clinical examination and/or provocative tests are positive and surgical management is being considered [70].

Ultrasonography

Recently, ultrasonography has gained more popularity in the diagnosis of CTS. An increase in the cross-sectional area of the median nerve has been correlated with the diagnosis of CTS. In their systematic review and meta-analysis of 97 observational studies published between 1992-2021 involving 6,679 wrists of healthy subjects, Asghar et al. revealed that the pooled estimate of the cross-section area of the median nerve at carpal tunnel inlet was 8.54mm2 (95% CI: 8.34-8.74mm2) [71]. The same pooled estimate at the carpal tunnel outlet was 8.03mm^2^ (95% CI: 7.46-8.60mm^2^). They suggested a reference value for median nerve disorders, including carpal tunnel syndrome: cross-section area of the median nerve at any place in carpal tunnel more than 10mm2 [71]. Ting et al. revealed a positive association between ultrasound measurement and electrodiagnostic studies in CTS patients [72]. Additionally, Wessel et al. revealed a positive association between the severity of CTS and increased cross-sectional area of the median nerve [73]. The advantages ultrasonography offers include patient comfort; lower cost; non-invasiveness; and its ability to rule out etiologies like mass lesions, tendinopathies, etc. However, local expertise is a requirement since ultrasound imaging is highly operator-dependent. Additionally, ultrasound cannot determine the severity of CTS or rule out etiologies such as polyneuropathies [53].

Management

Following the suspicion or the establishment of the diagnosis of CTS, primary care physicians should refer their patients to an orthopedic hand specialist. The management of CTS will depend on the severity of the disease. In mild and moderate cases of CTS, conservative treatment options are recommended. These include wrist splinting, local and systemic corticosteroids, non-steroidal anti-inflammatory drugs (NSAIDs), physical therapy, therapeutic ultrasound, and yoga [53]. Conservative management options encourage improved symptoms in two to six weeks and reach maximal benefits at three months [53,74]. Initially, patients are instructed on how to reduce their symptoms by modifying their movement at the wrist. Patients should practice proper hand ergonomics. Repetitive wrist movements should be avoided if possible.

A properly fitted splint that holds the wrist in a neutral position is widely recommended. Splinting is even more advisable for use in reversible cases of CTS, such as pregnancy, where it can be used to supplement other treatment options. Splints are low-cost, tolerable, simple, and effortless. A Cochrane review conducted in 2012 found nighttime wrist splinting to be more effective than placebo [75]. Additionally, the review found insufficient evidence to endorse one splint design over another or compare the effectiveness of splinting to other conservative treatment options.

Oral and local corticosteroids may be recommended for patients with mild to moderate CTS. Oral prednisone at a dosage of 20 mg daily for 10 to 14 days (about two weeks) was shown to be effective for the symptomatic treatment of CTS compared with a placebo, with its improvement lasting up to eight weeks [53,76-77]. However, oral corticosteroids are associated with some serious side effects, and they are less effective than corticosteroid injections. A 2007 Cochrane review reveals that local carpal tunnel corticosteroid injection produced symptomatic benefits for up to one month compared with placebo [76,78]. Ultrasound-guided injections are more effective and safer than blind injections [79]. Although local carpal tunnel corticosteroid injections are safe, misplaced injections can lead to median nerve injury and tendon rupture. Other medications like non-steroidal anti-inflammatory drugs, diuretics, and vitamin B6 are not effective, and evidence does not support their use [76,80-81].

Another conservative treatment option for mild to moderate CTS is physical therapy. Physical therapy includes carpal bone mobilization, therapeutic ultrasound, and nerve glide exercises. There is limited evidence to show that physical therapy is effective in the treatment of CTS [82,83]. Therapeutic ultrasound and carpal bone mobilization require an experienced therapist and several sessions [83]. Nerve glide exercises are quite simple and easy-to-learn exercises that can be done at home. Garfinkel et al. revealed that yoga offers some benefits in CTS compared to wearing a wrist splint [84].

Carpal tunnel release (CTR) surgery

Patients with severe CTS (such as when there is wasting of the thenar muscles or weakness of thumb opposition) or nerve damage (axonal degeneration) on nerve conduction studies should be offered surgical intervention with carpal tunnel release (surgical decompression). Patients should also be referred for surgical treatment when conservative measures fail or if the symptoms persist. CTR is usually performed by a neuro/orthopedic/plastic surgeon or hand surgeon. The procedure involves incising the transverse carpal ligament or flexor retinaculum, resulting in the decompression of the carpal tunnel, thus reducing the pressure on the median nerve. CTR has a lasting, good outcome in 70% to 90% of cases [85]. Furthermore, studies have shown that surgery is more effective and beneficial than conservative, non-surgical management options [86,87,88]. The surgical procedure can be performed either with an open approach or endoscopically. Although open CTR remains the traditional and most popular method of CTR, endoscopic CTR is gaining more popularity due to its more rapid recovery and improved safety profile [89]. Studies have shown that open and endoscopic CTR are equally effective; however, patients who had endoscopic CTR are able to return to work on average a week earlier than with open surgery [90,91].

## Conclusions

CTS is the most common compressive neuropathy of the upper limb. It costs the United States billions of dollars annually. The hallmark of typical CTS is pain, numbness, and paresthesia along the distribution of the median nerve. Conservative management and limitation of repetitive wrist movements are warranted in mild and moderate cases of CTS. Surgical decompression is effective for severe CTS, and it involves the release of the transverse carpal ligament to reduce the pressure on the median nerve within the carpal tunnel. Primary care physicians must understand this syndrome, so they can diagnose it and guide proper treatment.
